# Does TMS Disruption of the Left Primary Motor Cortex Affect Verb Retrieval Following Exposure to Pantomimed Gestures?

**DOI:** 10.3389/fnins.2018.00920

**Published:** 2018-12-12

**Authors:** Ana Murteira, Paul F. Sowman, Lyndsey Nickels

**Affiliations:** ^1^ARC Centre of Excellence in Cognition and its Disorders, Department of Cognitive Science, Macquarie University, Sydney, NSW, Australia; ^2^International Doctorate of Experimental Approaches to Language and Brain (IDEALAB), Macquarie University, Sydney, NSW, Australia; ^3^Perception in Action Research Centre, Faculty of Human Sciences, Macquarie University, Sydney, NSW, Australia

**Keywords:** action-verbs, gestures, priming, cTBS, interindividual variability

## Abstract

Previous research suggests that meaning-laden gestures, even when produced in the absence of language (i.e., pantomimed gestures), influence lexical retrieval. Yet, little is known about the neural mechanisms that underlie this process. Based on embodied cognition theories, many studies have demonstrated motor cortex involvement in the representation of action verbs and in the understanding of actions. The present study aimed to investigate whether the motor system plays a critical role in the behavioral influence of pantomimed gestures on action naming. Continuous theta burst stimulation (cTBS) was applied over the hand area of the left primary motor cortex and to a control site (occipital cortex). An action-picture naming task followed cTBS. In the naming task, participants named action pictures that were preceded by videos of congruent pantomimed gestures, unrelated pantomimed gestures or a control video with no movement (as a neutral, non-gestural condition). In addition to behavioral measures of performance, cTBS-induced changes in corticospinal activity were assessed. We replicated previous finding that exposure to congruent pantomimed gestures facilitates word production, compared to unrelated or neutral primes. However, we found no evidence that the left primary motor area is crucially involved in the mechanism underlying behavioral facilitation effects of gesture on verb production. Although, at the group level, cTBS induced motor cortex suppression, at the individual level we found remarkable variability of cTBS effects on the motor cortex. We found cTBS induction of both inhibition of corticospinal activity (with slower behavioral of responses) and enhancement (with faster behavioral responses). Our findings cast doubt on assumptions that the motor cortex is causally involved in the impact of gestures on action-word processing. Our results also highlight the importance of careful consideration of interindividual variability for the interpretation of cTBS effects.

## Introduction

The embodied cognition framework proposes that conceptual knowledge is grounded in interaction with the world and, therefore, that sensorimotor systems are an integral part of conceptual knowledge (e.g., Gallese and Lakoff, 2005; Barsalou, 2008).

Based on this framework, embodied approaches to language claim that, during processing of action-related words, the neural systems that are involved in forming and retrieving semantic knowledge overlap with the sensory modalities necessary for perceiving and/or for producing actions (e.g., Vigliocco et al., 2004; Fischer and Zwaan, 2008; Kiefer and Pulvermüller, 2012). Support for this theory comes from studies showing that motor-related areas of the brain are not only automatically engaged in tasks involving conceptual processing of action-related words (e.g., Oliveri et al., 2004; Repetto et al., 2013; Innocenti et al., 2014) but they also play a functional role in language processing (e.g., Pulvermüller et al., 2005; Vukovic et al., 2017; Courson et al., 2018). However, other studies have found evidence against specific engagement of the motor system in conceptual and lexical action word processing (e.g., de Zubicaray et al., 2013; Watson et al., 2013), suggesting that activity in the motor cortex might be secondary to, rather than strictly necessary for, action word processing. Mahon and Caramazza (2008) propose that semantic representations are amodal and activated in a semantic system that is outside the motor system but nonetheless, interact with sensory and motor information in the motor system. In other words, although independent systems, action and language might interact when sensorimotor activation is relevant, to enrich and complement the representation of a given concept. Taken together, the evidence seems to suggest that language and action should not be seen as isolated systems, but, at a minimum, functionally interactive.

An important expression of embodiment in cognition and in language are the hand gestures that speakers produce during communication. A variety of gestures are used in communication, but here we specifically address those gestures whose form conveys meaning that is related to the semantic content of the concept they represent (iconic gestures, e.g., moving the fingers in an inverted V-shape, representing the action of walking; McNeill, 1992; Özyürek, 2014). Research has shown that, when produced simultaneously with speech (i.e., co-speech iconic gestures), gestures influence cognitive processes such as learning, thought and language. For example, studies have found that co-speech iconic gestures and speech mutually interact to enhance listeners’ comprehension (Kelly et al., 2010, 2015) and that gesturing helps speakers retrieve words from the mental lexicon (Krauss et al., 2000). At the neuronal level, it has been suggested that the understanding of co-speech gestures, engages motor-related areas of the observer, in the same way as for the perception of human actions (e.g., Willems et al., 2009; Ping et al., 2014): Ping et al. (2014) found that the planning and production of arm and hand movements while watching a speaker producing co-speech gestures interfered with listeners’ abilities to interpret information from the gesture.

Altogether, this research provides an important contribution to the understanding of how gesture is intertwined with language processes and provides support for a degree of embodiment of cognitive processes (Hostetter and Alibali, 2008; Kita et al., 2017). Nevertheless, the focus of previous research has been on the study of gestures that naturally co-occur with speech. This leaves open questions about how other types of meaningful gestures may affect language processing and whether motor simulation could also play a role in the mechanisms underpinning the influence of these gestures on language.

Individuals can extract meaning from gestures even in the absence of speech, such as in the case of pantomimed gestures. Here, rather than using a strict definition of pantomime, we follow McNeill’s gesture continuum (McNeill, 1992, see also van Nispen et al., 2017 for a similar approach) and define pantomimed gestures as all meaningful gestures in which hand movements represent objects or actions that are understood in the absence of speech, and whose form is not fully conventionalized (e.g., re-enactment of the action of taking a telephone call with a closed fist by the ear). The relationship between pantomimed gestures and language could be seen as an example of language-motor interaction. As these gestures use the movement of hand and arms to represent the meaning of words, strong links could be expected between the elements of meaning (i.e., semantics) and action (i.e., the gesture). At the behavioral level, some studies indicate that pantomimed gestures can influence lexical processing in primed lexical decision (e.g., Wu and Coulson, 2007; Yap et al., 2011; So et al., 2013) or primed word reading (Bernardis et al., 2008; Bernardis and Caramelli, 2009). For example, So et al. (2013) found that the observation of pantomimed gestures primed visual lexical decision for semantically-related words and that the priming effect was stronger when gestures and speech were presented separately than when gestures co-occurred with speech. In speech production, little is known about the extent to which gesture may influence lexical retrieval. However, we (Murteira and Nickels, 2017; Murteira et al., in press) found faster response latencies for action picture naming when naming was preceded by the observation of a congruent pantomimed gesture, compared to when it was preceded by a meaningful but unrelated gesture. We hypothesized that one potential mechanism by which this priming could occur was from overlap in meaning (semantic representation) between the gesture and action. Similar effects were found in a study addressing the effects of pantomimes of tool-use on tool naming (Mounoud et al., 2007).

The neural mechanisms underpinning the relationship between pantomimed gestures and language production are poorly understood. Does motor simulation, as proposed for the comprehension and production of co-speech gestures (Hostetter and Alibali, 2008; Ping et al., 2014), play a crucial role in mediating the influence of pantomimed gestures on language production processes? Neuroimaging research has shown that gesture perception and understanding recruit an extensive and complex neuronal network (Villarreal et al., 2008; Andric and Small, 2012; Andric et al., 2013; Yang et al., 2015).

On the one hand, gestures represent meaning and therefore their comprehension activates brain regions involved in procssing semantic information. Previous findings have found strong activation of the left middle temporal region and inferior frontal gyrus from gestures (Villarreal et al., 2008; Andric et al., 2013; Yang et al., 2015), suggesting that these regions may be important for retrieval of semantic information and integration processes between gesture and speech (Yang et al., 2015).

On the other hand, gestures are actions and, as such, observing a gesture evokes similar neural responses to action recognition: studies have found that the observation of gestures induces strong activation in the bilateral motor-parietal network (Buccino et al., 2001; Villarreal et al., 2008; Quandt et al., 2012; Andric et al., 2013; Yang et al., 2015). Several neuroimaging and neurophysiological studies showed that the observation of actions recruits a network of precentral and parietal areas, including ventral and dorsal premotor cortex, inferior frontal gyrus, primary motor cortex, inferior parietal area and superior temporal cortex (Hari et al., 1998; Rizzolatti and Craighero, 2004; Fadiga et al., 2005; Caspers et al., 2010). The recruitment of motor-related areas in precentral and parietal regions during action observation is thought to reflect a causal role of the observer’s motor system in the understanding of actions (Rizzolatti and Sinigaglia, 2016). Similarities found between action production and gesture observation have been taken as evidence that sensorimotor systems are active when observing other people pantomiming object-related actions (Buccino et al., 2001; Quandt et al., 2012, but see Enticott et al., 2010). Moreover, other studies have found modulation of cortical oscillations in the alpha and beta rhythms in the primary motor cortex during observation of hand gestures (Quandt et al., 2012; Drijvers et al., 2018). However, specific involvement of the primary motor cortex was not found for the understanding of emblematic gestures (i.e., gestures with symbolic connotations, such as thumbs up gesture for “ok”), suggesting that the comprehension of these gestures might rely more strongly on neural circuits related to language processing (Campione et al., 2014).

In summary, behavioral evidence suggests that gesture and language are strongly linked even when the two systems do not overlap temporally (unlike the temporal overlap of co-speech gestures) and that the observation of gestures influences picture naming for nouns and verbs. However, little is known about the mechanisms that underlie this process. Whether motor simulation, resulting in motor cortex activation, is critical for gesture processing and action naming is still unclear.

One way to investigate this question is to use non-invasive brain stimulation, namely transcranial magnetic stimulation (TMS). To date, TMS studies have not investigated the role of the motor cortex in mediating the influence of gestures in language production.

Transcranial magnetic stimulation is a neurostimulation technique whereby neuronal activity is electrically stimulated by applying a transient magnetic field through the scalp. The electrical current generated, directly interferes with the ongoing electrical activity in the stimulated brain region of interest, temporarily modulating the dynamics of the neuronal population (Miniussi et al., 2013; Rotenberg et al., 2014). Therefore, a TMS-induced change in reaction time or accuracy can be used to infer potential causal relations between a specific brain region and a cognitive function (Robertson et al., 2003). A commonly used protocol to induce long-lasting modulation of brain activity is the application of repetitive trains of TMS (rTMS; Pascual-Leone et al., 2000). Several hypotheses have been suggested to explain the mechanisms by which TMS affects neuronal activity and the direction of behavioral effects (e.g., Pascual-Leone et al., 2000; Harris et al., 2008; Ruzzoli et al., 2010). Depending on the task demands, stimulation parameters and brain regions involved in the cognitive processes, rTMS may either increase or decrease cortical excitability (Hoogendam et al., 2010). In the past decade, a specific type of rTMS, continuous theta burst stimulation (cTBS), has gained attention due to its efficacy in inducing long-lasting depression of cortical excitability following short stimulation durations at low intensities (Huang et al., 2005; Goldsworthy et al., 2012; Wischnewski and Schutter, 2015; Chung et al., 2016). A common used cTBS paradigm is the application of short bursts of three TMS pulses at 50 Hz, with these bursts repeated at a frequency of 5 Hz and applied continuously for 40 s (Huang et al., 2005). Moreover, because, in cTBS, the stimulation is applied off-line, it minimizes the potential for confounds from non-specific stimulation effects that are inherent in on-line stimulation (e.g., scalp discomfort or muscle twitching; Sandrini et al., 2011). Nevertheless, recent studies indicate large intra- and inter-individual variability in the effects of cTBS-induced activity changes (Hamada et al., 2013; Vernet et al., 2014; Vallence et al., 2015; Heidegger et al., 2017). While the specific mechanisms accounting for this variability are still under investigation (see Ridding and Ziemann, 2010, for a review), some studies have shown that not all individuals exhibit cTBS-induced modulation of cortical activity, and some subjects even show paradoxical after-effects (Vernet et al., 2014; Heidegger et al., 2017).

### The Present Study

This study aimed to investigate the role that left primary motor cortex plays in the modulation of action naming performance by pantomime gesture observation. To this end, cTBS was used to suppress neural activity in the left primary motor area. The choice of this particular stimulation area is both theoretically and pragmatically-driven: (a) There is now extensive evidence that corticospinal activity in the motor system is modulated by action observation and action-word processing (although the functional contribution of this activation is still under debate); (b) under the embodied language hypothesis, the semantic representation of actions engages the premotor and motor systems; (c) there is strong reciprocal cortico-cortical connections linking the primary motor cortex and premotor areas (Matelli et al., 1986; Koch et al., 2010), hence stimulation in one area should indirectly affect the other; (d) stimulating motor cortex allows quantification of TMS-induced changes in corticospinal excitably. TMS motor evoked potentials (MEPs), recorded from peripheral hand muscles, can be measured using electromyography, hence providing an objective measure of corticospinal excitability (Rossini et al., 1998; Robertson et al., 2003). If the left primary motor cortex plays a role in facilitation of action naming by gestures, we would expect its temporary disruption to affect performance. The key outcome of interest was whether we observed an interaction between naming condition (congruent gesture, unrelated gesture, or no gesture prime) and stimulation (motor cortex, control site) in the effects on response latency and/or accuracy. If the left primary motor area is involved in processing of action-semantic aspects of meaning for both pantomimed gestures and words, its temporary disruption should affect the extent of facilitation of verb naming when those verbs are preceded by congruent gestures compared to unrelated gestures.

In addition to reaction time and accuracy measurements, corticospinal excitability, reflected in the size of TMS MEPs was measured pre- and post-stimulation.

To pre-empt the results, we found no effect of stimulation on response latencies or accuracy. Given the variability of motor cortex suppression shown by participants (and that is supported by the literature, e.g., Vallence et al., 2015), exploratory analyses were conducted to understand how this variability might have affected the results.

## Materials and Methods

### Participants

Thirty-two right-handed, English-native speakers (11 males), participated in the experiment. Participants’ age ranged from 18 to 40 years of age (*M*: 21 years, *SD*: 5 years). All presented with normal or corrected-to-normal vision and no reported history of psychological or neurological illness. Participants were screened for contraindications to TMS and gave informed consent for participation prior to the experiment. The study was approved by the Macquarie University Human Research Ethics Committee.

### Materials

Stimuli consisted of 72 black-and-white action pictures and color video-clips of pantomimed gestures. Action pictures were retrieved from a variety of sources (Bastiaanse, unpublished; Druks and Masterson, 2000; Szekely et al., 2004), and were included if the original source reported name agreement above 70% and Australian name agreement was above 80% (De Aguiar, 2015). For each action picture, a corresponding pantomimed gesture was created consisting of a 900 ms video clip of a woman miming an action using her arms and hands. Some gestures represented object-oriented actions (*n* = 41; e.g., drinking), whereas others represented non-object-oriented actions (*n* = 31; e.g., walking). Details of gesture stimulus preparation and normative data analysis can be found in Murteira (unpublished).

For the naming task, the action pictures (targets) were paired with gestures (primes) and presented in three conditions: (1) preceded by a congruent gesture (i.e., the gesture expressed the same action as the action depicted in the picture; e.g., *drinking_gest_* – drinking*_pic_*), (2) preceded by a unrelated gesture (i.e., the gesture expressed a different action to that depicted in the picture; e.g., *pushing_gest_* – drinking*_pic_*) or, (3) preceded by a neutral stimulus where no gesture was performed. Condition 3 served as a baseline condition for naming performance (i.e., the gesturer was shown in a static standing position and no gesture was performed).

In the unrelated condition, the gestures (as primes) and verbs (as targets) were recombined, making sure that the action represented by the gesture and the target verb were not associated (using the Edinburgh Associative Thesaurus; Kiss et al., 1973^1^), nor from the same verb class (using VerbNet; Kipper-Schuler, 2005; Palmer et al., 2017) and were not visually similar in body movement. Three sets of 24 gesture-action pairs were created and matched for relevant linguistic properties of the verb depicted in the picture, including: verb lemma log frequency (SUBTLEX-UK; Van Heuven et al., 2014), imageability (where available, Cortese and Fugett, 2004), number of phonemes (MRC Psycholinguistic Database, version 2.00), transitivity, instrumentality and visual complexity (De Aguiar, 2015). Hence, participants named a target action picture only once per session, but item presentation systematically varied within each subset such that, across participants and sessions, all target action pictures were named in all conditions.

### Stimulation and Recording

#### Single Pulse TMS

Individual changes in corticospinal excitability were assessed with single-pulse TMS-MEPs. First, single-pulse TMS (Magstim Super Rapid, Magstim, Whitland, United Kingdom) was delivered over the left primary motor cortex to determine the optimal site for MEP elicitation and to determine resting and active motor thresholds for each participant. A 70 mm figure-of-eight coil was orientated at 45 degrees to the scalp with current flowing posterior-anterior across the primary motor cortex. Coil position and angle was adjusted until the optimal site for consistent elicitation of MEPs was identified. Once the optimal site for stimulation was located, this was marked on the scalp. Then, resting motor threshold was determined by identifying the minimal single-pulse TMS intensity necessary to elicit a MEP from the right first dorsal interosseous muscle, while the hand was at rest, with a peak-to-peak amplitude of 50 μV in 5 out of 10 consecutive stimulations (Rothwell et al., 1999; Sandrini et al., 2011). Activity from the right first dorsal interosseous muscle was recorded by surface electromyography (1000x gain, bandpass filters from 0.3 to 1000 Hz) from Ag–AgCI electrodes with bipolar electrode montage. The electromyogram (EMG) was amplified using an ADInstruments dual bio-amp, digitized via an ADInstruments PowerLab 8/30 and controlled by LabChart 7 (ADInstruments).

Participants’ resting motor thresholds ranged from 46 to 80% of the maximum stimulator output (*M* = 62, *SD* = 8).

Single-pulse TMS-MEPs were recorded at three different time points: (1) before cTBS (i.e., baseline); (2) 5 min post-stimulation, immediately before the naming task (Time 1) and (3) at the end of naming task (Time 2). A reduction in MEP amplitude after cTBS over the left primary motor cortex was used as indicator of induced corticospinal inhibition (Wischnewski and Schutter, 2015).

Participants’ individual active motor threshold were also recorded to set the intensity for cTBS. Active motor threshold was defined as the minimum single pulse TMS intensity necessary to elicit MEPs, in the right first dorsal interosseous muscle, with peak-to-peak amplitude greater than 200 μV in 5 out of 10 consecutive trials while participants maintain a voluntary hand contraction of about 20% of the maximal voluntary contraction (Rothwell et al., 1999; Sandrini et al., 2011). Participants’ active motor threshold ranged from 38 to 76% of the maximum stimulator output (*M* = 53, *SD* = 7.5).

#### Continuous Theta Burst Stimulation

Continuous theta burst stimulation was administered, using a Magstim Rapid2 system (Magstim, Whitland, United Kingdom) and a 70-mm figure-of-eight coil, over the left primary motor cortex or control site stimulation (occipital pole), following Huang and colleagues’ protocol (Huang et al., 2005). Each cTBS burst consisted of three pulses at 50 Hz, with bursts repeated at a frequency of 5 Hz, applied continuously for 40 s and delivered at an intensity of 80% of active motor threshold (Huang et al., 2005). Whenever a participant’s 80% of active motor threshold exceeded 51% (this was the case for two participants), theta burst intensity was kept at 51% as this is the maximum stimulator output intensity at 50 Hz. cTBS was delivered over the left primary motor cortex at a mean intensity of 42.3% (*SD* = 5).

##### Control site stimulation

The control site was defined as the location of electrode Oz on the international 10–20 system of scalp electrodes. The coil was positioned approximately midline of the occipital cortex and held with the handle pointing upward. This site has been previously used as a control site in studies investigating the neural substrates of semantic representation of objects (e.g., Pobric et al., 2010; Ishibashi et al., 2011; Chiou et al., 2014) and no adverse reactions have been reported.

### Procedure

Each participant completed two sessions, 1 week apart, receiving either cTBS over the left primary motor cortex or cTBS over the control site. The order of sessions was counterbalanced across participants. At the start of the session, individual resting and active motor thresholds were measured. Following measurement of the motor thresholds, the naming task was explained, and participants performed the practice trial. Based on the individual resting motor threshold values, two blocks of 20 MEPs were recorded at 120% of the resting motor threshold. The average of these two blocks was used as the baseline MEP measurement. Then, cTBS was applied for 40 s, followed by a 5-min pause, in which participants were instructed to remain at rest and relaxed. This duration of waiting time, prior to beginning the naming task, was chosen based on previous findings regarding cTBS after-effects, which have shown greater MEP modulation 5 min post-stimulation (Vernet et al., 2014), compared to TMS-induced MEPs measured immediately after stimulation. After the waiting time, the first post-stimulation block of 20 MEPs was recorded, immediately followed by the naming task. Directly after naming task completion, the second post-stimulation block of 20 MEPs was recorded.

The control session followed the same sequence of events, except that stimulation was applied over the control site (occipital pole). MEPs were measured from the primary motor cortex during the control session as a measure of individual motor cortex excitability stability.

### Naming Task

The naming task was implemented in Presentation^®^software (Version 16.3^2^). A single trial comprised: (i) a fixation cross appearing in the center of the screen for 1000–2000 ms; (ii) a gesture or no movement video clips for 900 ms; (iii) a short fixation cross appearing in the center of the screen for 100 ms; (iv) an action picture for 2000 ms. Participants were instructed that video clips of gestures followed by pictures of actions would appear on the screen. They would be asked to name the pictures of actions, as quickly and accurately as possible, using a single verb in the *-ing* form, but no spoken response (covert or overt) should be given in response to the video clips. Each participant performed six practice trials and 72 experimental trials (24 target action pictures per condition). The order of stimulus presentation was randomized across participants. Vocal responses were recorded via an external microphone and reaction time was measured from picture display onset. Responses were manually checked with Audacity^®^software 2.1.1^3^ to ensure accurate vocal reaction time measurement.

### Analysis

#### Motor Evoked Potentials (MEPs)

Changes in MEP amplitudes were expressed as a percentage of pre-stimulation baseline levels for descriptive analysis of the data. Participants whose changes in MEP amplitude were greater than 2.5 SD from the group mean were considered outliers and removed from further data analysis (*n* = 3). Baseline pre-stimulation MEPs were averaged for each subject prior to analysis. Comparison between cTBS over the primary motor cortex and control site on the changes in MEP amplitudes after cTBS was performed with a two-way ANOVA with Site (motor cortex, control site) and Time (post-stimulation Time 1, post-stimulation Time 2) as within-subject factors (for this analysis, MEP amplitudes were expressed as a percentage of baseline). Statistical analysis on the changes in MEP amplitudes after cTBS over the primary motor area was performed with a one-way ANOVA on raw data with Time (pre-cTBS stimulation, post-stimulation Time 1, post-stimulation Time 2) as a within subject factor. Paired samples *t*-tests were used to determine at which time points MEP amplitudes were significantly different from pre-stimulation.

#### Effect of Motor Cortex Stimulation on Naming Task Performance

For naming latency analysis, incorrect responses^4^, no-responses, and trials with reaction times greater than 2.5 SD from each condition’s mean (2.6% in total) were excluded from analysis. A Box and Cox test (Box and Cox, 1964; Osborne, 2010) indicated that it was appropriate to use a log-transformation on the reaction time data.

Naming latencies were analyzed with linear mixed-effects modeling (Baayen et al., 2008), computed using the lme4 package (Bates et al., 2015b) in the R environment (RStudio Team, 2016). Significance testing of fixed effects was performed by likelihood ratios (χ^2^) for model comparison (Pinheiro and Bates, 2000). Whenever a fixed effect or an interaction between fixed effects was significant, the appropriate contrasts were computed using the multcomp package (Hothorn et al., 2008). The significance of a model’s fixed parameter estimates was estimated with Satterthwaite approximations of degree of freedom using the lmerTest package (Kuznetsova et al., 2017). A summary of the models’ parameter estimates can be found in Supplementary Material. If the model with an interaction between the fixed effects explained significantly more variance than the model with just the main effects, we report the interaction model. Otherwise, the simpler model is reported. The model random effects structure was determined by a principal component analysis (PCA) and stepwise model comparison of goodness of fit (χ^2^), which aimed to identify the random effects structure best supported by the data (Bates et al., 2015a; Matuschek et al., 2017).

Accuracy was analyzed by computing a generalized linear mixed model for binomial data (Baayen et al., 2008) from the lme4 package (Bates et al., 2015b). Significance of fixed effects, fixed effects estimates, and random effects structure selection were performed following the same procedures as described for naming latencies.

## Results

### Motor Evoked Potential Amplitude Changes Pre- to Post-cTBS as an Indicator of Corticospinal Excitability Modulation

**FIGURE 1:**
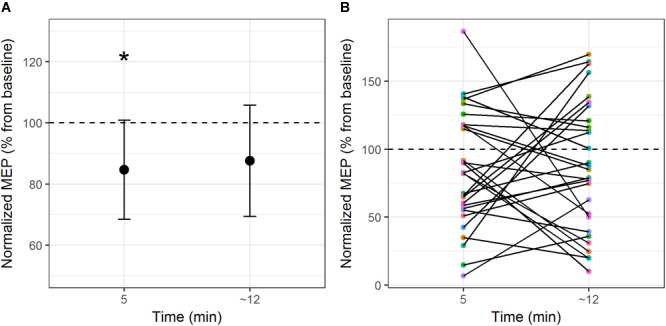
**(A)** Mean MEP modulation (expressed as percentage change of baseline) from pre-stimulation to post-stimulation over the primary motor cortex at the group level. **(B)** Individual subject MEP modulation at post-stimulation Time 1 and post-stimulation Time 2. ^∗^*p* < 0.05 (two-tailed).

The comparison between cTBS over the primary motor cortex and control site on MEP amplitude demonstrated a main effect of Site (*F*_1,28_ = 4.4, *p* = 0.04, η^2^= 0.14). Motor evoked potential amplitudes were significantly lower during stimulation over the motor cortex compared to stimulation over the control site (with a mean difference of 28% of baseline MEP amplitude). There was no significant main effect of Time (*F*_1,28_ = 0.6, *p* = 0.45, η^2^= 0.02) nor an interaction between Time and Site (*F*_1,28_ = 0.02, *p* = 0.88, η^2^= 0.001). A one-way repeated measures ANOVA was computed to compare differences in MEP amplitudes pre- and post-cTBS on the primary motor cortex, with Time as independent variable (three levels: baseline; Time 1–5 min post-stimulation; Time 2 – end of the naming task). Results showed a significant effect of Time on MEP amplitude (*F*_2,56_ = 3.4, *p* = 0.04, η^2^= 0.11). Compared to pre-stimulation, MEP amplitudes were significantly lower at post-stimulation Time 1 [*t*(28) = 2.5, *p* = 0.017 (two-tailed), *d* = 0.47; mean difference = 0.23 ± 0.09 mV] and marginally significant at post-stimulation Time 2 [*t*(28) = 1.99, *p* = 0.057 (two-tailed), *d* = 0.37; mean difference = 0.20 ± 0.1 mV]. Figure 1A illustrates the time course of mean changes in MEP amplitudes (expressed as a percentage of baseline) following the cTBS protocol. It should be noted that, although stimulation-induced change in corticospinal excitability was significant at the group level 5 min post-stimulation, individual participant responses were highly variable. As depicted in Figure 1B, 69% of the participants showed reduced MEP amplitudes at 5 min post-stimulation. 52% of the participants maintained MEP amplitude reduction at the end of the naming task.

### Effect of cTBS on Naming Performance (Whole Group Analysis)

The linear mixed-effects model for the naming latency analysis included log-transformed Reaction time as the dependent variable and fixed effects of Condition, Stimulation and the interaction between Condition and Stimulation. The model intercept was set to represent performance in the neutral, control site stimulation condition (i.e., the baseline condition) and variables’ coefficients were compared to this intercept. As participants were exposed to the same naming task over 2 days (i.e., cTBS control site session and cTBS motor cortex session), Session was included as a covariate in the model to account for any possible influence of this variable. Random-effects structure selection procedures resulted in a model that included by-participants and by-items random intercepts, by-participants random slopes for Condition and Stimulation site and by-items random slopes for Condition. Table 1 displays the mean reaction times (in ms) by naming condition and stimulation site. A summary of the model parameter estimates can be found in Supplementary Material (Table A1).

**Table 1:** Mean response latency for each naming condition in each stimulation site.

	cTBS control site				cTBS motor cortex			
	Response latency (ms)				Response latency (ms)			
	95% CI of the mean				95% CI of the mean			
Condition	Mean	*SD*	Lower	Upper	Mean	*SD*	Lower	Upper
Congruent	833	108	792	875	842	121	796	888
Unrelated	944	112	901	986	959	138	906	1011
Neutral	951	112	909	994	944	117	899	988

**FIGURE 2:**
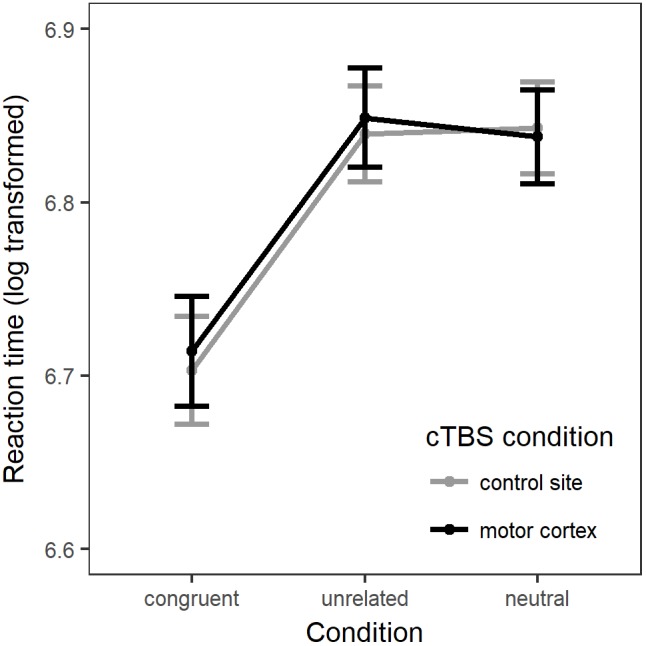
The linear mixed model’s fitted values for response latency (log transformed). Errors bars = standard error of the model fit.

There was a significant main effect of Condition [χ^2^(2) = 44.5, *p* < 0.001]. Compared to the neutral (baseline) condition, action naming was significantly faster when preceded by congruent gestures (*b* = -0.13, *SE* = 0.02, *z* = -8.42, *p* < 0.001), but there was no significant difference in response time when naming was preceded by unrelated gestures (*b* = 0.004, *SE* = 0.01, *z* = 0.42, *p* = 0.91). There was no main effect of Stimulation [χ^2^(1) = 0.17, *p* = 0.68], and including the interaction between Condition and Stimulation did not significantly improve the model fit [χ^2^(2) = 1.66, *p* = 0.44; see Figure 2].

**FIGURE 3:**
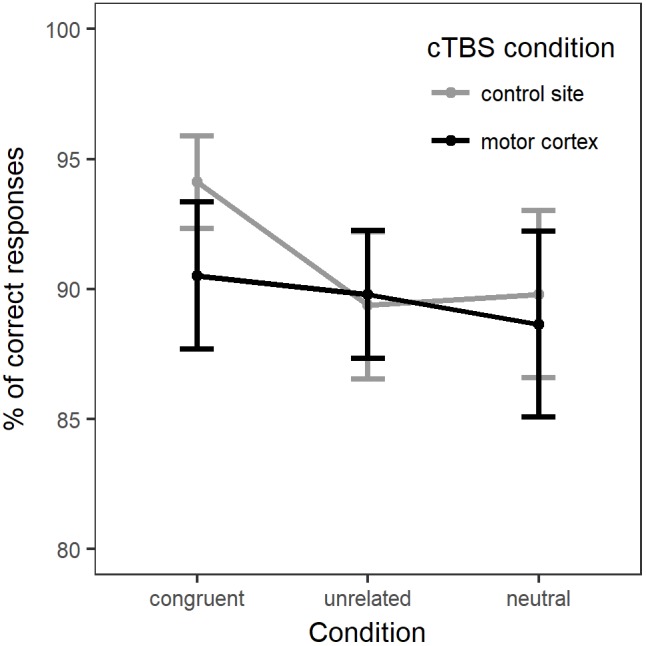
Percentage of correct responses for each naming condition in each stimulation site (whole group).

For the analysis of naming accuracy, the generalized linear mixed-effects model included accuracy as a dependent variable and the same fixed effects: Naming Condition, Stimulation Site, their interaction and Session. Once again, the model intercept represented performance in the neutral, control site stimulation condition. Random-effects structure selection procedures resulted in a model that included by-participants and by-items random intercepts and by-items random slopes for Condition. Figure 3 displays the percentage of correct responses across naming conditions and stimulation sites. In Supplementary Material (Table A2) provides a summary of the model parameter estimates.

There was no main effect of Condition [χ^2^(2) = 0.12, *p* = 0.94] or of Stimulation [χ^2^(1) = 2.47, *p* = 0.12]. Adding the interaction between Condition and Stimulation only marginally improved the fit of the model [χ^2^(2) = 5.25, *p* = 0.07]. Comparison between the models’ Akaike Information Criteria (AIC) showed that the model with the interaction term had the lower AIC (model with interaction: AIC = 2249.5; model without interaction: AIC = 2250.8), hence providing the best fit to the data (Akaike, 1974). Therefore, we computed *post hoc* comparisons between Naming condition and Stimulation (*p*-values adjusted with Holm–Bonferroni correction). Interaction contrasts indicated only a marginal effect of cTBS stimulation on accuracy between naming in the congruent condition and naming in the unrelated condition (*b* = -0.33, *SE* = 0.14, *z* = -2.35, *p* = 0.057). The contrasts between neutral versus related or unrelated conditions under motor cortex stimulation compared to control stimulation did not reach significance.

One might question the rationale for stimulating the hand area only, as not all gestures represented hand-related actions. According to several studies, a somatotopic organization of action representations in the motor cortex (e.g., Buccino et al., 2001; Hauk et al., 2004) exists, although this is yet to be specifically investigated with meaningful communicative gestures. To address this potential issue, a *post hoc* analysis was conducted to discriminate between possible effects of hand area stimulation on hand-related actions. This analysis included the interaction between Hand-related action and Condition and Stimulation Site. We found no interaction between Hand-related actions and Stimulation Site or Condition [χ^2^(5) = 1.7, *p* = 0.89]. There was, however a main effect of hand relatedness, as non-hand-related actions were named faster than hand-related actions [χ^2^(1) = 14.8, *p* = 0.0001], but no main effect of Stimulation Site [χ^2^(1) = 0.18, *p* = 0.67]. These results found no effect of stimulation even when distinctions are made between hand-related and non-hand-related actions. A summary of the model parameter estimates is provided in Supplementary Material.

### Exploring the Effect of Motor Evoked Potential Modulation as a Possible Index of Motor Cortex Suppression

As noted above, the effect of cTBS on MEP amplitude modulation was extremely variable. This variability raised the possibility that the effects of motor cortex stimulation on the action naming task may have varied across participants. Hence, we conducted further exploratory analyses to determine whether induced cortical activity, as reflected in MEP amplitude modulation, influenced the effects found: the interaction between stimulation site (cTBS of motor cortex; cTBS of control site) and MEP amplitude change from pre-stimulation to post-stimulation was included in the mixed-effect model for the analyses of response latency and accuracy (e.g., model with main effects of Condition and Stimulation). For each of the dependent variables, two models were computed: the first included the MEP amplitude changes at Time 1 (i.e., 5 min after stimulation and immediately before naming task) relative to baseline MEP amplitude; the second included the MEP amplitude changes at Time 2 (i.e., immediately after naming task) relative to baseline MEP amplitude. The resulting models were compared to models without the interaction term, by likelihood ratio tests, while keeping the remaining fixed and random-effects structure unaltered. See Supplementary Material (Table A3) and A.4 for a summary of the models.

**FIGURE 4:**
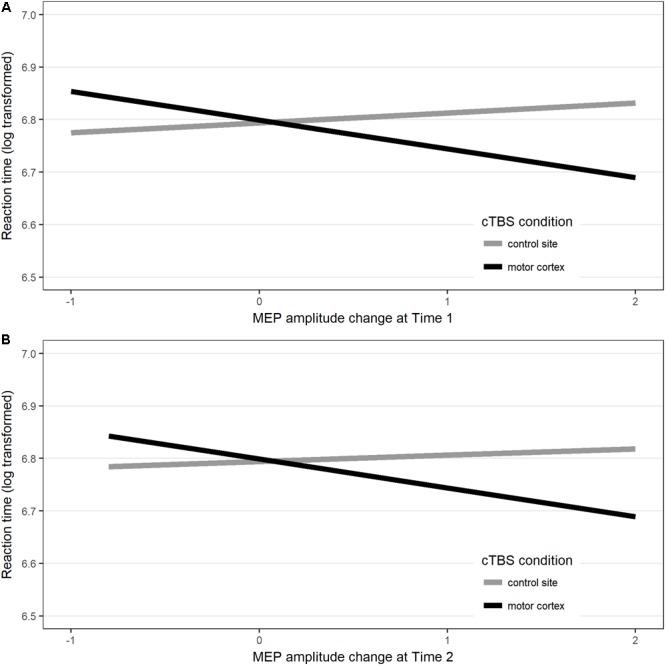
Interaction between response latency (values represent the linear mixed model’s fitted values for response latency) in each Stimulation site and MEP amplitude changes measured at **(A)** post-stimulation Time 1 and **(B)** post-stimulation Time 2 compared to baseline.

Results showed that, for response latency, including the interaction between stimulation site and post-stimulation MEP amplitude change at Time 1 only marginally increased the fit of the model [χ^2^(1) = 3.47, *p* = 0.062]. Comparison between the models’ AICs (Akaike, 1974) showed that the model with the interaction had the lower AIC (model with interaction: AIC = -2078.1; model without interaction: AIC = -2076.6), hence providing the best fit to the data. In this model, the fixed parameter estimates showed a significant interaction between motor cortex cTBS and MEP amplitude change (*b* = -0.073, *SE* = 0.036, *t* = -2.05, *p* = 0.0495). Interestingly, this effect seemed to be driven by the fact that, when cTBS was applied to the motor cortex, compared to control site stimulation, naming latencies were faster in participants who showed greater increase of MEP amplitude at Time 1 relative to baseline, (i.e., those participants who showed an unexpected paradoxical after-effect of the cTBS application which is normally expected to produce MEP suppression). There was no main effect of stimulation, nor a main effect of MEP amplitude change at Time 1 [see Figure 4A and in Supplementary Material (Table A3)].

Including the interaction between stimulation and MEP amplitude changes at Time 2 marginally enhanced the fit of the model [χ^2^(1) = 3.13, *p* = 0.077]. This result is in line with the fact that, compared to pre-stimulation baseline motor cortex excitability, no significant motor cortex suppression was found when MEPs were measured at the end of the naming task. Nevertheless, a comparison between the model’s AIC (Akaike, 1974) showed that the model with the interaction had the lowest AIC (model with interaction AIC = -2078.2; model without interaction AIC = -2077.1), hence providing the best fit to the data. At the end of the naming task, after cTBS had been applied to the motor cortex, there was a trend for participants who showed increased MEP amplitude, relative to baseline, to have faster naming latencies than after control site stimulation, although the effect was not statistically significant [*b* = -0.076, *SE* = 0.038, *t* = -1.75, *p* = 0.09; see Figure 4B and in Supplementary Material (Table A3)].

For accuracy, including the interaction between stimulation and MEP amplitude change did not significantly affect the fit of the model at Time 1 [χ^2^(1) = 0.507, *p* = 0.477], nor at Time 2 [χ^2^(1) = 2.407, *p* = 0.121; see Supplementary Material (Table A4)].

Based on the results showing a significant effect of the interaction between cTBS on primary motor cortex and MEP amplitude change on response latency, we further explored the results (for both response latencies and accuracy) with the subset of participants who showed MEP suppression post-stimulation.

### Results for Participants Showing Motor Evoked Potential Suppression Following Motor Cortex Stimulation

This exploratory analysis included only the 15 participants who showed a numerical decrease in MEP amplitude post-stimulation. The aim of these analyses was to explore the possibility that an interaction between Condition and Stimulation site might only be evident in those participants who showed MEP suppression following motor cortex stimulation. Incorrect responses, no-responses, and trials with reaction times greater than 2.5 SD from each condition’s mean (2.6% in total) were excluded from analysis. Response latency analysis followed the same procedure as described for the whole group analysis. The final statistical models included the same fixed and random effects structure as for the whole group analyses. Table 2 displays the mean reaction times (in ms) by naming condition and stimulation site and the model parameter estimates are summarized in Supplementary Material (Table A5).

**Table 2:** Mean response latency for each naming condition in each stimulation site for the subgroup of participants who showed MEP amplitude reduction post-stimulation (*n* = 15).

	cTBS control site				cTBS motor cortex			
	Response latency (ms)				Response latency (ms)			
	95% CI of the mean				95% CI of the mean			
Condition	Mean	*SD*	Lower	Upper	Mean	*SD*	Lower	Upper
Congruent	839	133	765	912	852	102	795	909
Unrelated	949	132	876	1022	978	129	906	1049
Neutral	955	109	895	1015	961	118	896	1027

The results for naming latency followed the same pattern as for the whole group analysis. There was a main effect of Condition [χ^2^(2) = 26.41, *p* < 0.001], but no main effect of Stimulation [χ^2^(1) = 1.814, *p* = 0.178], nor a significant interaction between Condition and Stimulation [χ^2^(2) = 1.60, *p* = 0.449]. Pairwise comparisons of estimated marginal means for each naming condition in each stimulation site are present in Table 3. There was no significant effect of motor cortex stimulation across naming conditions. As for the whole group analysis, a supplementary analysis was conducted to discriminate between possible effects of hand area stimulation on hand-related actions. There was no interaction between Hand-related actions and Stimulation Site or Condition [χ^2^(5) = 2.2, *p* = 0.82]. Non-hand-related actions were named faster than hand-related actions [χ^2^(1) = 14.2, *p* = 0.0002], but there was no main effect of Stimulation Site [χ^2^(1) = 1.8, *p* = 0.18]. Again, these results find no impact of stimulation even when distinctions are made between hand-related and non-hand-related actions for participants who showed MEP suppression following motor cortex stimulation. A summary of the model parameter estimates is provided in Supplementary Material.

**Table 3:** Response latency: pairwise comparisons of the estimated marginal means for each naming condition in each stimulation site for the subgroup of participants who showed MEP amplitude reduction post-stimulation (*n* = 15).

Contrasts	Estimate	*SE*	t.ratio	Lower CL	Upper CL	*p*-value^a^
CS, mat – MC, mat	–0.030	0.021	–1.418	–0.072	0.012	0.47
CS, mis – MC, mis	–0.031	0.021	–1.451	–0.073	0.011	0.44
CS, neutral – MC, neutral	–0.009	0.021	–0.436	–0.051	0.033	1

*^*a*^Bonferroni correction; CS = control site stimulation; MC = motor cortex stimulation; lower CL = asymptotic confidence limit; upper CL = upper asymptotic confidence limit.*

**FIGURE 5:**
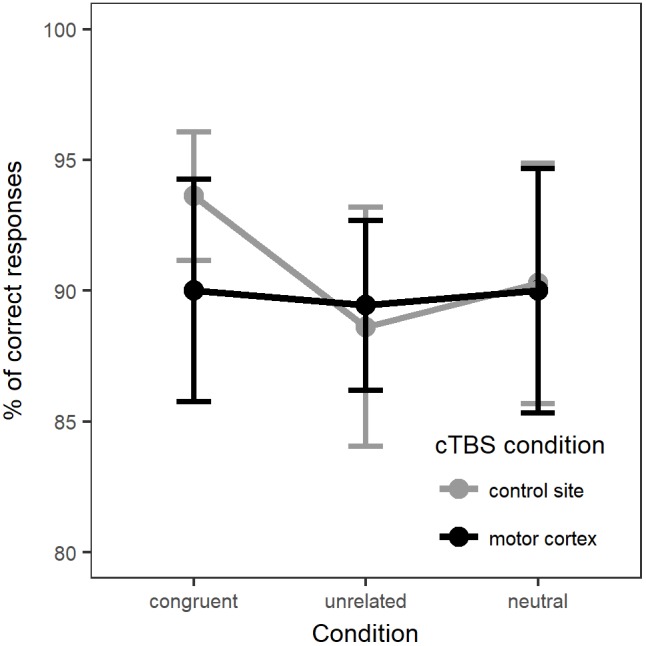
Percentage of correct responses for each naming condition at each stimulation site (15 participants with MEP amplitude suppression).

Accuracy was analyzed using generalized mixed-effects modeling, following the same procedure as described for the whole group analysis. The final model included the same fixed and random effects structure as for the whole group analysis. Figure 5 displays the percentage of correct responses across naming conditions and stimulation sites. A summary of the model parameter estimates can be found in Supplementary Material (Table A6).

The results for naming accuracy diverged from the pattern found for the whole group analysis. There was a main effect of Stimulation [χ^2^(1) = 4.90, *p* = 0.027], with less accurate responses for motor cortex stimulation compared to control site stimulation (*b* = -0.359, *SE* = 0.163, *z* = -2.204, *p* = 0.028). There was no main effect of Condition [χ^2^(2) = 0.47, *p* = 0.790]. Adding the interaction between Condition and Stimulation did not significantly improve the fit of the model [χ^2^(2) = 2.58, *p* = 0.274]. Pairwise comparisons of estimated marginal means for each naming condition in each stimulation site are present in Table 4. These comparisons suggested a possible effect of motor cortex suppression in the congruent condition, however, the threshold for statistical significance was not met after correction for multiple comparisons of the estimated marginal means. Due to the exploratory nature of this analysis and the small sample size, further investigation would be required to confirm this result.

**Table 4:** Accuracy: pairwise comparisons of the estimated marginal means for each naming condition in each stimulation site for the subgroup of participants who showed MEP amplitude reduction post-stimulation (*n* = 15).

Contrasts	Estimate	*SE*	z.ratio	Lower CL	Upper CL	*p*-value^a^
CS, mat – MC, mat	0.716	0.299	2.392	0.129	1.303	0.05
CS, mis – MC, mis	0.067	0.275	0.244	–0.473	0.607	1
CS, neutral – MC, neutral	0.337	0.275	1.225	–0.202	0.876	0.66

*^*a*^Bonferroni correction; Results are given on the log (not response) scale; CS = control site stimulation; MC = motor cortex stimulation; lower CL = asymptotic confidence limit; upper CL = upper asymptotic confidence limit.*

## Discussion

Observation of pantomimed gestures has been demonstrated to facilitate naming of the actions that they represent (Murteira and Nickels, 2017; Murteira et al., in press). Based on theories of embodied cognition (e.g., Barsalou, 2008), previous work on co-speech gestures has suggested that the listener’s motor system is involved in comprehending gestural information (Ping et al., 2014). Drawing on this embodied perspective, a large number of studies have found changes in corticospinal excitability during lexical processing of action words (e.g., Pulvermüller et al., 2005; Repetto et al., 2013). Moreover, studies have shown sensorimotor cortex activation during gesture observation (e.g., Andric et al., 2013; Yang et al., 2015). If motor simulation is indeed central to the integration of gesture and language, then temporary disruption of the hand area of the left primary motor cortex should interfere with the behavioral effects of gesture observation on action naming.

To test this hypothesis, we used cTBS, a TMS protocol that produces relatively sustained neuronal modulation peaking at around 5 min following stimulation (Huang et al., 2005). cTBS was applied to the hand area of the left primary motor cortex and over a control site in the occipital region. After stimulation, participants named action pictures following observation of congruent or unrelated pantomimed gestures. In addition to behavioral outcome measures (i.e., reaction time and accuracy performance), individual changes in corticospinal excitability were assessed with TMS-induced MEPs, recorded prior to and twice post-stimulation.

We replicated our previous finding that observation of a congruent pantomimed gesture facilitates action-picture naming, relative to an unrelated or a neutral prime. However, critically, cTBS to the hand area of the left primary motor cortex did not significantly interfere with action naming performance, nor was there a significant interaction between suppression of the motor cortex and pantomimed gesture prime type. Even when we investigated the subgroup of participants who showed reduced motor cortex excitability post-stimulation, the data suggested no significant interaction between pantomime prime type and stimulation for response latency, although there was a decrease in response accuracy after cTBS of the motor cortex on the congruent condition, which may be worth further investigation. Consequently, this experiment provides no evidence to support a crucial role for the left primary motor cortex in the neural mechanisms underlying the facilitatory effect of gesture on verb production.

As Miniussi and Thut (2010) pointed out, one difficulty in interpreting why a cognitive task is unaffected by TMS to a given brain region, is that there may be multiple brain regions participating in the task, such that interfering with neural activity in one region does not induce a major change in behavior. Previous studies have shown engagement of the motor cortex when people observe hand actions (Buccino et al., 2001; Enticott et al., 2010; Quandt et al., 2012) and during conceptual processing of action-words (e.g., Oliveri et al., 2004; Pulvermüller et al., 2005; Innocenti et al., 2014). Consequently, it seemed reasonable to expect that this region would be active when observing pantomimed gestures. However, unlike many co-speech gestures, pantomimed gestures have clear semantic content and can be understood in the absence of co-occurring speech. This suggests that they should have a closer relationship with language processing, likely involving a fronto-temporal network for semantic processing (Villarreal et al., 2008; Willems et al., 2009; Xu et al., 2009). Without disregarding the evidence for motor cortex activation during gesture observation, our data suggest that the left primary motor cortex may not be involved in the integration of gesture with language (at least in the facilitation of lexical retrieval), given that suppression of this region, at the group level, did not affect behavioral effects of gesture observation on action naming. This result is in line with findings from neuroimaging research demonstrating that conceptual processing of action only inconsistently involve premotor and motor areas (Watson et al., 2013) This result may be problematic for motor simulation accounts of gesture and action-word processing (Barsalou, 2008; Ping et al., 2014). However, it is in line with Campione et al. (2014) study, who demonstrated that emblematic gestures (i.e., symbolic gestures with conventionalized forms) and their corresponding words did not involve activation of the left primary motor cortex, as measured by evaluating TMS-induced MEPs. Hence, the integration of information conveyed by a gesture with a specific semantic representation, which in turn facilitates the lexical representation of a related word, might primarily involve core language areas, rather than the motor cortex. An alternative explanation is that, within the action/gesture observation network, we cannot exclude a more essential role for other areas in action understanding compared to the primary motor cortex. Neuroimaging studies have consistently shown the involvement of premotor areas during action observation (for a review see Caspers et al., 2010). If we assume motor theories of action understanding are correct, the simulation account of action representations could primarily involve premotor areas and, hence, stimulating only motor cortex might not have been enough to yield an effect. This could be a limitation of the study. However, it should be noted that, in our experiment, cTBS after-effects in fact induce corticospinal changes that modulated the behavioral responses – we just did not find an interaction between stimulation and action-naming priming by the observation of congruent gestures. There also exists a broad literature demonstrating strong reciprocal cortico-cortical connection between the motor and premotor cortices (Matelli et al., 1986; Kakei et al., 2001; Dum and Strick, 2005; see Kilner and Frith, 2007, for a discussion about primary motor activation during action observation), hence, in our study, an interaction between stimulation of the motor cortex and activity in premotor areas cannot be discarded. In future research, the use of combined neurostimulation and neuroimaging could help better interpret the strength of focal stimulation effects and at the same time disentangle how the connected networks are affected by stimulation (Hallett et al., 2017; Keysers et al., 2018; Polanía et al., 2018).

An important aspect of our experiment that is worth discussion, is the variability of cTBS effects we observed between participants. One advantage of stimulating over the primary motor cortex is that post-stimulation corticospinal excitability can be directly measured by single pulse TMS-induced MEPs. In the present study, we followed Huang and colleagues’ cTBS protocol (Huang et al., 2005) in order to suppress cortical activity in the primary motor cortex. This TMS inhibitory protocol is widely used due to its supposed robust and long-lasting effects (Chung et al., 2016). Our results showed that, at the group level, motor cortex suppression could, in fact, be induced, but the effect was smaller, and the suppressed duration shorter, than has been previously reported in the literature (e.g., Huang et al., 2005, but see Goldsworthy et al., 2012). Critically, participants’ responses to the stimulation were highly variable. Not all participants presented with the expected post-stimulation reduction of MEP amplitude, and some demonstrated modulation of cortical activity in the opposite direction (i.e., 31% of the participants showed either no variation or increased MEP amplitude following stimulation). We are not alone in finding variability. These results are in line with other recent reports showing highly variable response patterns following cTBS (e.g., Vallence et al., 2015; Heidegger et al., 2017; Hordacre et al., 2017). For example, Heidegger et al. (2017), in a study with 31 healthy participants, found depressive effects of cTBS in only 19% of the participants, and paradoxical responses in 23% of the participants.

With these patterns of response variability in mind, we further investigated whether modulation of cortical excitability post-cTBS influenced the direction of the behavioral effects. For response latencies, we found a significant interaction between cTBS application to the motor cortex and MEP amplitude changes immediately post-stimulation. The source of the interaction primarily arose from those individuals with MEP amplitudes that were increased compared to baseline and these were associated with faster naming response latencies, compared to control site stimulation. We also found the expected slower response latencies from individuals with decreased MEP amplitudes post-stimulation as a result of the predicted cortical inhibition from cTBS. In other words, in this experiment, cTBS after-effects induced both corticospinal inhibition (and consequently slower behavioral responses) and corticospinal facilitation (and consequently faster behavioral responses). The effects induced by cTBS of the motor cortex affected naming response latency in general, however no relationship was found between priming of verb naming by the observation of congruent gestures and stimulation.

Behavioral facilitation in language tasks following cTBS has been previously reported (e.g., Willems et al., 2011; Bonnì et al., 2015). However, in these studies, cTBS was applied in brain regions outside the motor cortex and it was not possible to obtain a measure of cortical excitability modulation. Hence, it was hard to establish associations between the direction of neuronal activity and the behavioral outcomes. In our study it seems that, for some participants, cTBS enhanced cortical excitability, which in turn facilitated naming performance.

There is compelling evidence that the decrease of cortical excitability induced by cTBS is due to long term depression (LTD)-like mechanisms (Hoogendam et al., 2010). However, the complex relationship between these mechanisms, synaptic activity of the stimulated region, and genetic and physiological factors that underpin interindividual variability in the size, duration and direction of cTBS effects, is not fully understood (Siebner and Rothwell, 2003; Ridding and Ziemann, 2010; Vallence et al., 2015). One hypothesis that has been put forward to explain the interindividual variability obtained with single-pulse TMS on cognitive function, and that could also be useful to understand paradoxical effects obtained with repetitive TMS, is that improved or impaired performance in a cognitive task might dependent on the relationship between signal and noise in the stimulated area (Miniussi et al., 2013; Bonnì et al., 2015). The activity induced by TMS changes the ratio between the neural population that codes for the stimulus (i.e., signal) and other irrelevant neural activity that is unrelated to the task (i.e., noise). The noise induced by TMS interacts with the relevant neural activity and will affect the relationship between activated and non-activated neurons and the final behavioral response (see Miniussi et al., 2013 for a review on the topic). Several factors that can influence the neuronal activity of the stimulated area and, consequently, neuroplastic responses to TMS (Ridding and Ziemann, 2010 for a review) have been identified. In addition to stimulation parameters, external factors, including the time of the day that stimulation occurs or prior motor activity (e.g., Sale et al., 2007; Gentner et al., 2008) are thought to contribute to interindividual variability. Although we tried to control for effects of these potential confounds, we are not able to completely exclude the possibility that they may have had some influence. Experimental sessions were, as much as possible, scheduled for the afternoon and at the same time across the two sessions, however, this was not always possible. Previous studies have suggested that prior motor activation, such as the voluntary muscle contraction necessary to assess the active motor threshold, modulates cTBS outcomes (Goldsworthy et al., 2012; Vernet et al., 2014), but evidence to adjudicate on this contention is mixed (e.g., Huang et al., 2005). Nevertheless, motor activity prior to the stimulation was carefully controlled during the experiment. In addition, during the waiting period that followed the application of cTBS, participants were requested to remain relaxed and, at the same time, surface electromyographic activity was monitored to measure changes in cortico-spinal excitability.

Our experiment demonstrates that despite a carefully controlled design, response variability to changes in corticospinal excitability is almost inevitable, and that there is still uncertainty about the extent to which experimental variables and other external factors modulate neural responses to TMS. Research using TMS to study cognitive functions needs to acknowledge the presence of this interindividual variability, particularly in paradigms where it is not possible to obtain an objective measurement of the direction of cortical activity (e.g., studies on language production).

## Conclusion

The present study extends our understanding of the interactions between the gestural and the word production systems, providing further evidence for gestural priming of action verbs.

However, the results have implications for previous literature that emphasizes a causal role of the motor cortex in the processing of gestures and action-words. In our study, facilitation of action naming by pantomimed gestures did not seem to involve the primary motor cortex. Hence, contrary to what has been proposed in the literature for the integration of co-speech gestures and speech, pantomimed gesture processing does not seem to necessarily rely on motor simulation mechanisms (assuming motor simulation involves the motor cortex).

An important aspect of this study is that, in addition to latency and accuracy, our methodology allowed us to measure cortical excitability and thereby observe how the motor system was modulated post-stimulation. We found that cTBS induced both inhibition and enhancement of corticospinal activity, which in turn, impaired and improved overall naming performance, respectively. Hence, our results provide further evidence for large interindividual variability in the response to cTBS at both the level of motor cortex excitability and at the level of behavioral measurement.

Transcranial magnetic stimulation is a useful tool to investigate the relationship between cortical areas and cognitive functions. However, transcranial stimulation research needs to acknowledge and thoroughly assess interindividual variability, in order to avoid potentially misleading results. Ultimately, it is only by a greater understanding of such variability that we will be able to develop better experimental designs and ensure that our research results are accurate, robust, and replicable.

## Author Contributions

AM, PS, and LN conceived and designed the experiment, analyzed and interpreted the data, and wrote the manuscript. AM carried out the experiment. All authors approved the final version of the submitted manuscript.

## Conflict of Interest Statement

The authors declare that the research was conducted in the absence of any commercial or financial relationships that could be construed as a potential conflict of interest.

## Footnotes

1 http://www.eat.rl.ac.uk

2 http://www.neurobs.com

3 http://www.audacityteam.org/

4 Synonyms and direct troponyms (e.g., *drink* – sip) were accepted as correct. Analyses considering such responses as incorrect gave the same results.
