# Supplementary material for: Does TMS Disruption of the Left Primary Motor Cortex Affect Verb Retrieval Following Exposure to Pantomimed Gestures?
Source: Front Neurosci. 2018 Dec 12;12:920. doi: 10.3389/fnins.2018.00920 (PMC6299802; doi:10.3389/fnins.2018.00920)
Supplement: Supplementary file 1 [file Data_Sheet_1.docx]

**Appendix A:** Linear Mixed Effect model summaries

Table A.1. Response latency: Summary of the linear mixed-effects model estimates (simple effects of Condition and Stimulation against the intercept)

| R Model equation: RT ~ Condition + Stimulation + (1+ Condition + Stimulation \| Participant) + (1 + Condition \| Item) | | | | | |
| --- | --- | --- | --- | --- | --- |
| Fixed effects | β | SE β | t-value | p-value |  |
| (intercept: neutral; control site) | 6.837 | 2.637e-02 | 259.310 | < .001 |  |
| Condition congruent | -1.319e-01 | 1.583e-02 | -8.33 | **< .001** |  |
| Condition unrelated | 3.735e-03 | 9.148e-03 | 0.41 | .686 |  |
| Motor cortex stimulation | 5.210e-03 | 1.293e-02 | 0.40 | .690 |  |
| Session (covariate) | 3.452e-02 | 6.435 e-03 | -5.36 | < .001 |  |
| Random effects | Variance | SD | Corr. |  |  |
| Items (intercept) | 0.013 | 0.11 |  |  |  |
| Condition congruent | 0.005 | 0.07 | -0.05 |  |  |
| Condition unrelated | 0.001 | 0.04 | 0.01 | 0.24 |  |
| Participants (intercept) | 0.014 | 0.12 |  |  |  |
| Condition congruent | 0.004 | 0.06 | 0.12 |  |  |
| Condition unrelated | 0.0005 | 0.02 | 0.11 | 0.27 |  |
| Motor cortex stim. | 0.004 | 0.06 | -0.23 | 0.06 | 0.41 |
| Residual | 0.028 | 0.17 |  |  |  |

Table A.2. Accuracy: Summary of the generalised linear mixed-effects model estimates (simple effects of Condition, Stimulation and interaction between Conditions and Stimulation against the intercept)

| R Model equation: Accuracy ~ Condition * Stimulation + (1 \| Participant) + (1 + Condition \| Item) | | | | |
| --- | --- | --- | --- | --- |
| Fixed effects | β | SE β | z-value | p-value |
| (intercept: neutral; control site) | 3.258 | 0.308 | **10.567** | **< .001** |
| Condition congruent | 0.259 | 0.297 | 0.870 | .384 |
| Condition unrelated | -0.193 | 0.282 | -0.686 | .493 |
| Motor cortex stimulation. | -0.131 | 0.190 | -0.691 | .489 |
| Congruent*Motor cortex stim. | -0.450 | 0.284 | -1.585 | .113 |
| Unrelated* Motor cortex stim. | 0.214 | 0.267 | 0.804 | .421 |
| Session (covariate) |  |  |  |  |
| Random effects | Variance | SD | Corr. |  |
| Items (intercept) | 3.13 | 1.77 |  |  |
| Condition congruent | 0.733 | 0.86 | -0.75 |  |
| Condition incongruent | 0.781 | 0.88 | -0.37 | 0.77 |
| Participants (intercept) | 0.355 | 0.60 |  |  |

Table A.3. Summary of the linear mixed-effects model estimates for the model including the interaction between Stimulation and MEP modulation at Time 1 and the model including interaction between Stimulation and MEP modulation at Time 2

| R model equation: RT ~ Condition + Stimulation + MEP*Stimulation + (1+ Condition + Stimulation \| Participant) + (1+Condition \| Item) | | | | | | | | | | |
| --- | --- | --- | --- | --- | --- | --- | --- | --- | --- | --- |
|  | MEP amplitude change Time 1 | | | |  | MEP amplitude change Time 2 | | | |  |
| Fixed effects | β | SE β | t-value | p-value |  | β | SE β | t-value | p-value |  |
| (intercept) | 6.837 | 2.644e-02 | 258.527 | < .001 |  | 6.837 | 2.651e-02 | 257.882 | < .001 |  |
| Condition congruent | -1.132e-01 | 1.586e-02 | **-8.312** | **< .001** |  | -1.318e-01 | 1.582e-02 | -8.332 | **< .001** |  |
| Condition unrelated | 3.803e-03 | 9.099e-03 | 0.418 | .679 |  | 3.825e-03 | 9.155 e-03 | 0.418 | .679 |  |
| Motor cortex stim | 5.218e-03 | 1.241e-02 | 0.421 | .678 |  | 4.878 e-03 | 1.250e-02 | 0.390 | .700 |  |
| MEPs | 1.888e-02 | 1.107e-02 | 0.896 | .378 |  | 1.212 e-02 | 1.805 e-02 | 0.671 | .507 |  |
| Session (covariate) | 3.753 e-02 | 6.526 e-03 | 5.751 | < .001 |  | 3.841 e-02 | 6.690 e-03 | 5.741 | < .001 |  |
| stim*MEPs | -7.353e-02 | 3.359e-02 | **-2.049** | **.050** |  | -6.710e-02 | 3.836e-02 | -1.749 | **.090** |  |
| Random effects | Variance | SD | Corr. |  |  | Variance | SD | Corr. |  |  |
| Items (intercept) | 0.013 | 0.12 |  |  |  | 0.013 | 0.12 |  |  |  |
| Condition congruent | 0.005 | 0.07 | -0.05 |  |  | 0.005 | 0.07 | -0.05 |  |  |
| Condition unrelated | 0.001 | 0.04 | 0.01 | 0.24 |  | 0.001 | 0.04 | 0.01 | 0.24 |  |
| Participants (intercept) | 0.014 | 0.12 |  |  |  | 0.014 | 0.12 |  |  |  |
| Condition congruent | 0.004 | 0.06 | 0.18 |  |  | 0.004 | 0.06 | 0.16 |  |  |
| Condition unrelated | 0.0005 | 0.02 | 0.13 | 0.26 |  | 0.0005 | 0.02 | 0.13 | 0.27 |  |
| Motor cortex stim | 0.004 | 0.05 | -0.21 | -0.13 | 0.14 | 0.004 | 0.06 | -0.23 | 0.05 | 0.44 |
| Residual | 0.028 | 0.17 |  |  |  | 0.028 | 0.17 |  |  |  |

Table A.4. Accuracy: Summary of the generalised mixed-effects model estimates for the model including the interaction between Stimulation and MEP modulation at Time 1 and the model including interaction between Stimulation and MEP modulation at Time 2

| R model equation: Accuracy~ Condition + Stimulation + MEP*Stimulation + (1+ Condition + Stimulation \| Participant) + (1 + Condition\| Item) | | | | | | | | | | |
| --- | --- | --- | --- | --- | --- | --- | --- | --- | --- | --- |
|  | MEP amplitude change Time 1 | | | |  | MEP amplitude change Time 2 | | | |  |
| Fixed effects | β | SE β | z-value | p-value |  | β | SE β | z-value | p-value |  |
| (intercept) | 3.291 | 0.298 | 11.044 | < .001 |  | 3.293 | 0.299 | 11.020 | < .001 |  |
| Condition congruent | -3.06e-03 | 0.247 | -0.012 | < .99 |  | -3.21e-03 | 0.247 | -0.013 | 0.990 |  |
| Condition unrelated | -8.316e-02 | 0.247 | -0.337 | .736 |  | 0.083 | 0.247 | -0.337 | 0.736 |  |
| Motor cortex stim | -0.189 | 0.113 | -1.676 | .094 |  | -0.177 | 0.113 | -1.561 | 0.119 |  |
| MEP | -0.115 | 0.185 | 0.623 | .534 |  | -0.015 | 0.163 | -0.090 | 0.928 |  |
| Session (covariate) | -0.239 | 0.060 | -3.952 | < .001 |  | -0.255 | 0.062 | -4.123 | < .001 |  |
| stim*MEP | 0.221 | 0.300 | 0.735 | .462 |  | 0.499 | 0.314 | 1.590 | 0.112 |  |
| Random effects | Variance | SD | Corr. |  |  | Variance | SD | Corr. |  |  |
| Items (intercept) | 3.13 | 1.77 |  |  |  | 3.15 | 1.77 |  |  |  |
| Condition congruent | 0.73 | 0.86 | -0.76 |  |  | 0.74 | 0.86 | -0.77 |  |  |
| Condition unrelated | 0.78 | 0.88 | -0.37 | 0.77 |  | 0.79 | 0.87 | -0.37 | 0.77 |  |
| Participants (intercept) | 0.35 | 0.59 |  |  |  | 0.36 | 0.60 |  |  |  |

Table A.5. Response latency: Summary of the linear mixed-effects model estimates (simple effects of Condition and Stimulation and interaction between Conditions and Stimulation effects against the intercept)

| R Model equation: RT ~ Condition + Stimulation + (1+ Condition + Stimulation \| Participant) + (1 + Condition \| Item) | | | | | |
| --- | --- | --- | --- | --- | --- |
| Fixed effects | β | SE β | t-value | p-value |  |
| (intercept: neutral, control site) | 6.842 | 3.442e-02 | 198.748 | < .001 |  |
| Condition congruent | -1.349e-01 | 1.765e-02 | -7.645 | **< .001** |  |
| Condition unrelated | 3.773e-03 | 1.765e-02 | 0.267 | .793 |  |
| Motor cortex stim. | 2.352e-02 | 1.693e-02 | 1.390 | .185 |  |
| Session (covariate) | 3.455 e-02 | 8.118 e-03 | 4.256 | .001 |  |
| Random effects | Variance | SD | Corr. |  |  |
| Items (intercept) | 0.014 | 0.12 |  |  |  |
| Condition congruent | 0.005 | 0.07 | -0.04 |  |  |
| Condition unrelated | 0.003 | 0.05 | -0.19 | 0.48 |  |
| Participants (intercept) | 0.014 | 0.12 |  |  |  |
| Condition congruent | 0.002 | 0.05 | 0.36 |  |  |
| Condition unrelated | 0.001 | 0.03 | -0.10 | -0.40 |  |
| Motor cortex stim. | 0.003 | 0.06 | -0.27 | -0.25 | 0.38 |
| Residual | 0.03 | 0.17 |  |  |  |

Table A.6. Accuracy: Summary of the generalised linear mixed-effects model estimates (simple effects of Condition and Stimulation)

| R Model equation: Accuracy ~ Condition * Stimulation + (1 \| Participant) + (1 + Condition \| Item) | | | | |
| --- | --- | --- | --- | --- |
| Fixed effects | β | SE β | z-value | p-value |
| (intercept; neutral, control site) | 3.281 | 0.362 | 9.052 | < .001 |
| Condition congruent | 0.044 | 0.377 | 0.117 | .907 |
| Condition unrelated | 0.253 | 0.401 | 0.632 | .527 |
| Motor cortex stim. | -0.359 | 0.162 | **-2.204** | **.028** |
| Session (covariate) | -0.216 | 0.081 | -2.654 | .008 |
| Random effects | Variance | SD | Corr. |  |
| Items (intercept) | 3.281 | 1.618 |  |  |
| Condition congruent | 0.859 | 0.927 | -0.54 |  |
| Condition unrelated | 0.710 | 0.843 | 0.28 | 0.66 |
| Participants (intercept) | 0.232 | 0.481 |  |  |
